# Comorbidities and treatment patterns in adult patients with atopic dermatitis: results from a nationwide multicenter study

**DOI:** 10.1007/s00403-021-02243-w

**Published:** 2021-06-07

**Authors:** A. Campanati, T. Bianchelli, R. Gesuita, C. Foti, G. Malara, G. Micali, P. Amerio, F. Rongioletti, M. Corazza, A. Patrizi, K. Peris, N. Pimpinelli, A. Parodi, M. C. Fargnoli, S. P. Cannavo, P. Pigatto, G. Pellacani, S. M. Ferrucci, G. Argenziano, F. Cusano, G. Fabbrocini, L. Stingeni, M. C. Potenza, M. Romanelli, L. Bianchi, A. Offidani, and collaborators, P. Romita, M. L. Musumeci, V. Piras, A. Borghi, C. Loi, N. Gori, F. Prigano, R. Gallo, M. Esposito, A. Campitello, L. Bolzano, S. Tavecchio, G. Calabrese, L. Di Costanzo, K. Hansel, N. Skroza, E. Tolino, G. Tonini, M. Talamonti

**Affiliations:** 1 Department of Clinical and Molecular Sciences, Dermatology Unit, Polytechnic Marche University, Ancona, Italy; 2 Centre of Epidemiology and Biostatistics, Polytechnic Marche University, Ancona, Italy; 3 Department of Biomedical Science and Human Oncology, Dermatological Clinic, University of Bari, Bari, Italy; 4 Department of Dermatology Grande, Ospedale Metropolitano “Bianchi Melacrino Morelli”, Reggio Calabria, Italy; 5 Dermatology Clinic, University of Catania, PO G. Rodolico, AOU Policlinico-Vittorio Emanuele, Catania, Italy; 6 Department of Medicine and Aging Science, Dermatology Unit, University G.D’Annunzio Chieti, Chieti, Italy; 7 Vita-Salute - S, Raffaele University, Milan, Italy; 8 Department of Medical Sciences, Dermatology Unit, University of Ferrara, Ferrara, Italy; 9 Department of Experimental, Diagnostic and Specialty Medicine, Division of Dermatology, University of Bologna, Bologna, Italy; 10 Fondazione Policlinico Universitario A. Gemelli IRCCS, Dermatologia, Rome, Italy; 11 Sezione di Dermatologia, Dipartimento di Scienze Mediche e Chirurgiche, Università Cattolica del Sacro Cuore, Rome, Italy; 12 Oncologia Medica, Università Cattolica del Sacro Cuore, Rome, Italy; 13 Department Health Science Section of Dermatology, University of Florence, Florence, Italy; 14 Dermatology Clinic, Ospedale Policlinico San Martino, Largo R. Benzi, 10, 16132 Genoa, Italy; 15 Dermatology, Department of Biotechnological and Applied Clinical Sciences, University of L’Aquila, L’Aquila, Italy; 16 Dermatology Unit, University Hospital Policlinico “G. Martino”, Messina, Italy; 17 Clinical Dermatology, IRCCS Istituto Ortopedico Galeazzi, Milan, Italy; 18 Dermatology Unit, Department of Surgical, Medical, Dental and Morphological Science With Interest Transplant, Oncological and Regenerative Medicine, University of Modena and Reggio Emilia, Modena, Italy; 19 Department of Physiopathology and Transplantation, Università Degli Studi Di Milano, Milan, Italy; 20 Dermatology Unit, Fondazione IRCCS Ca’ Granda Ospedale Maggiore Policlinico, Via Pace, 9, 20122 Milan, Italy; 21 Dermatology Unit, University of Campania, Naples, Italy; 22 Unit of Dermatology, G. Rummo Hospital, Benevento, Italy; 23 Department of Clinical Medicine and Surgery, Section of Dermatology, University of Naples Federico II, Naples, Italy; 24 Dermatology Section, Department of Medicine, University of Perugia, Perugia, Italy; 25 Dermatology Unit, Department of Medico-Surgical Sciences and Biotechnologies, Faculty of Pharmacy and Medicine, Sapienza University of Rome - Polo Pontino, Rome, Italy; 26 Dermatology Unit, Department of Clinical and Experimental Medicine, University of Pisa, Pisa, Italy; 27 Dermatology Unit, Policlinico Tor Vergata Rome, University of Rome Tor Vergata, Rome, Italy

**Keywords:** Adult atopic dermatitis, Epidemiology, Comorbidity, Treatment, Immunosuppressants, Biologics

## Abstract

Adult atopic dermatitis (adult AD) is a systemic inflammatory disorder, whose relationship with immune-allergic and metabolic comorbidities is not well established yet. Moreover, treatment of mild-to-moderate and severe atopic dermatitis needs standardization among clinicians. The aim of this study was to evaluate the distribution of comorbidities, including metabolic abnormalities, rhinitis, conjunctivitis, asthma, alopecia and sleep disturbance, according to severity of adult AD, and describe treatments most commonly used by Italian dermatologists. Retrospective, observational, nationwide study of adult patients over a 2-year period was performed. Clinical and laboratory data were obtained through review of medical records of patients aged ≥ 18 years, followed in 23 Italian National reference centres for atopic dermatitis between September 2016 and September 2018. The main measurements evaluated were disease severity, atopic and metabolic comorbidities, treatment type and duration. Six-hundred and eighty-four adult patients with AD were included into the study. Atopic, but not metabolic conditions, except for hypertension, were significantly associated with having moderate-to-severe AD in young adult patients. Disease duration was significantly associated with disease severity. Oral corticosteroids and cyclosporine were the most widely used immunosuppressant. Our study seems confirm the close relationship between adult AD and other atopic conditions, further long-term cohort studies on patients affected by adult AD need to be performed to evaluate the complex relationship between adult AD disease severity and metabolic comorbidities.

## Introduction

Atopic dermatitis (AD) usually appears in early childhood (15–30%) and generally resolves prior to puberty. However, in up to half of patients, it may persist into adulthood, becoming a lifelong condition [1, 2].

Although prevalence of adult atopic dermatitis (adult AD) remains unclear, several studies have indicated that it has been increasing in recent decades, particularly in industrialized countries [3]. Current estimates place the prevalence of AD at around 2–8% in adults, compared with 10–20% in children [4, 5].

Adult AD is considered a systemic and immune-allergic inflammatory skin disease, and it was assumed that atopic (allergic rhinitis, asthma), metabolic comorbidities (increased body mass index (BMI), central obesity, hypertension, hyperlipidaemia, diabetes mellitus) and sleep disturbance can promote the chronic inflammatory state, capable of perpetuating the progression of the disease, leading to more severe forms of adult AD [6, 7].

Topical and systemic corticosteroids are the cornerstone of pharmacological treatment regardless disease severity [8, 9].

However, in moderate and severe cases, several systemic treatments are used in clinical practice, including cyclosporine, which is the only one approved for treatment of adult AD, followed by methotrexate, azathioprine, and mycophenolate mofetil.

There are still no population-based Italian studies on the prevalence of comorbidities in adult AD based on the severity of disease.

Similarly, the need for observational studies describing therapeutic interventions that reflect the impact on these patients is growing. Aims of this study were to evaluate the distribution of associated adult AD comorbidities according to disease severity, through the stratification of patients with mild, moderate and severe disease, and describe treatments prescribed for adult patients in routine clinical practice, according to disease severity.

## Materials and methods

### Study design

This is a multicenter observational study, conducted in accordance with the latest revision of the Helsinki Declaration (2009/58). All enrolled patients had signed an informed consent to make their clinical data available for research purposes. The study was approved by Local Ethical Committee United Hospital of Ancona.

### Population

Data from 684 patients (356 males and 328 females) with mild-to-severe atopic dermatitis, aged ≥ 18 years, in treatment with conventional topical and systemic drugs at 23 Dermatological clinical centres of Italian National Health System were retrospectively collected from outpatients and inpatients medical records from September 2016 to September 2018 and recorded into an electronic medical record.

### Clinical collected data

Information such as demographic data (age and sex), anthropometric measurements (height, weight and BMI, waist and hip circumference), metabolic (hypertriglyceridemia, hypercholesterolemia, hypertension, diagnosis of type 2 diabetes mellitus), atopic (rhinitis, asthma, conjunctivitis) comorbidities and functional impairment as sleep disorders (including difficulty falling asleep, frequent night time awakenings, and excessive daytime sleepiness) and pruritus were retrieved. Previous and on-going treatments for each patient were collected as well.

### Clinimetric tools

Severity of adult AD for each subject was recorded as mild, moderate or severe, according to Eczema Area and Severity Index (EASI) and SCORing AD (SCORAD). Quality of life of patients were investigated through the Dermatology Life Quality Index (DLQI) questionnaire.

EASI is a validated investigator-assessed scoring system that grades the physical signs of atopic dermatitis, it determines the severity of the patient’s eczema, according to clinicians’ perspective [10], its final score ranges from 0 to 72.

SCORAD index is a mixed patient/clinicians tool to evaluate AD severity [11], its final score ranges from 0 to 103, and determines disease severity according to patients and clinicians’ perspective.

Diagnosis and disease severity of atopic dermatitis was established according Italian AD guidelines [12], AD diagnosis was based essentially on the disease typical clinical signs and symptoms, evaluated by experienced dermatologists, as currently no diagnostic markers are available. Disease severity was graduated as follows: mild disease EASI < 16 and/or SCORAD < 25; moderate disease EASI =  > 16 and/or SCORAD > 25; severe disease EASI =  > 21 and/or SCORAD > 50, or EASI score < 16 but at least one of the following conditions: itch with numeric rating scale (NRS) score > 7 and/or sleep disturbances with NRS score > 7.

DLQI is a simple, self-administered and user-friendly validated questionnaire designed to assess the impact of a wide range of skin disease on patient health-related quality of life [13]. Final total score is calculated by summing the score of ten items which cover six domains including symptoms and feelings, daily activities, leisure, work and school, personal relationships, resulting in a maximum score of 30 and a minimum score of 0. The higher scores the poorer the quality of life.

Pruritus was quantified through The Peak Pruritus Numerical Rating Scale (NRS) a validated, single-item, patient-reported outcome (PRO) of itch severity. Patients were asked to rate the intensity of their itch using a visual analogue scale rating from 0 ("no itch") to 10 (“worst imaginable itch”).

### Statistical analysis:

A non-parametric approach was used since variables had a no-normal distribution, when evaluated with the Shapiro test. Quantitative variables were summarized using median and interquartile range (IQR, 1^st^–3rd quartiles), respectively, as measure of centrality and variability; qualitative variables were expressed as absolute and percent frequencies. Comparisons between groups were evaluated using Wilcoxon rank sum test and Fisher exact test, respectively.

A logistic regression analysis was performed to evaluate demographic and clinical factors associated with the probability of having a severe vs mild/moderate AD.

Likelihood Ratio (LR) test was used to detect the variables to be included in the model and models’ goodness of fit was evaluated by the Hosmer and Lemeshow (HL) test. All estimates were evaluated as 95% confidence intervals (95%CI).

## Results

A total of 684 adult patients with AD were included in this study, among which 79 (11.5%) were classified to have mild, 71 (10.5%) moderate, and 534 (78%) severe AD. Distribution of demographic, anthropometric and clinical characteristics of the patients according to adult AD severity is summarized in Table 1.

**Table 1:** Demographic, anthropometric and clinical characteristics of the patients according to adult AD severity

			Adult AD		
		Mild	Moderate	Severe	*p*
Gender [*n* (%)]	M	41 (51.9)	30 (42.3)	285 (53.4)	0.212*
	F	38 (48.1)	41 (57.7)	249 (46.6)	
AGE [median (IRQ)]	Years	30 (24;48)	29 (24;40)	37 (26;50)	0.005
		Moderate vs Severe			
Disease duration [median (IRQ)]	Years	18 (6;27)	18 (7;27)	23 (15;35)	< 0.001
		Mild and moderate vs Severe			
DLQI [median (IRQ)]	(0–30)	4 (2;6)	7 (4;12)	16 (11;21)	< 0.001
		Mild vs Moderate and Severe; Moderate vs Severe			
Pruritus [mediana (IRQ)]	(1–10)	3 (2;5)	5 (2;7)	8 (7;10)	< 0.001
		Mild vs Moderate and Severe; Moderate vs Severe			
BMI [median (IRQ)]	Kg/m^2^	23 (21;25)	23 (21;27)	24 (21;26)	0.301
Waist circumference [median (IRQ)]	Cm	75 (68;90)	73 (68;83)	81 (72;90)	0.004
		Moderate vs Severe			
Hip circumference [median (IRQ)]	Cm	92 (86;100)	91 (84;96)	94 (86;100)	0.466
Glycaemia [median (IRQ)]	mg/dL	84 (78;90)	88 (78;91)	80 (72;89)	0.004
		Moderate vs Severe			
Serum cholesterol median (IRQ)]	mg/dL	180 (160;202)	180 (169;198)	170 (153;196)	0.069
Serum triglycerides [median (IRQ)]	mg/dL	90 (76;110)	109 (87;138)	94 (76;125)	0.061
Serum cholesterol HDL [median (IRQ)]	mg/dL	56 (50;68)	52 (47;59)	54 (46;61)	0.097
Systolic blood pressure [median (IRQ)]	mmHg	120 (110;125)	120 (119;125)	120 (110;130)	0.804
Dyastolic blood pressure [median (IRQ)]	mmHg	80 (70;80)	78 (70;80)	80 (70;80)	0.587
W/H (males)		0.54 (0.50; 0.58)	0.53 (0.45; 0.55)	0.53 (0.49; 0.57)	0.482
W/H (females)		0.56 (0.53; 0.60)	0.56 (0.53; 0.58)	0.56 (0.51; 0.60)	0.865

*p* values refer to Kruskal–Wallis for quantitative variables; IRQ: 1°–3° quartiles

*Chi-square test for categorical variables

Patients having severe diseases were significantly older than the other (*p* = 0.05), and no difference in adult AD severity according to sex between groups was evident (*p* = 0.212).

Median disease duration ranging from 18 years (IQR: 6–27 for mild adult AD, and 7–27 for moderate adult AD) to 23 years (IQR: 15–35) for severe AD, with significantly longer lasting disease for patients having severe adult AD (*p* < 0.001).

Impact of adult AD on quality of life was significantly different across the three groups, with DLQI gradually increasing from mild 4 (IQR: 2–6) to moderate 7 (IQR: 4–12) and severe 16 (IQR: 11–21) atopic dermatitis (*p* < 0.001).

Pruritus (NRS peak pruritus) showed the same pattern, and it was more pronounced in severe Adult AD (8; IQR: 7–10), compared to moderate (5; IQR: 2–7) and mild (3; IQR: 2–5).

Among metabolic features (Tables 1and2), hypertension was found significantly more frequent in the severe adult AD group (*p* < 0.001); no significant difference was retrieved across the three groups for: BMI (*p* = 0.301), abdominal obesity (considered as W/H > 0.9 for males and 0.85 for females; *p* = 0.999), hypercholesterolemia (*p* = 0.658), hypertriglyceridemia (*p* = 0.602), diabetes mellitus (*p* = 0.841).

**Table 2:** Metabolic comorbidities according to adult AD severity

		Adult AD				
		Mild	Moderate	Severe		*p*
		*n* = 79	*n* = 71	*n* = 534	*n* = 684	
		*n* = 69	*n* = 62	*n* = 378	*n* = 509	
Hypercholesterolemia [*n* (%)]	No	51 (73.9)	48 (77.4)	298 (78.8)		0.658
	Sì	18 (26.1)	14 (22.6)	80 (21.2)	112 (22)	
		*n* = 69	*n* = 62	*n* = 374	*n* = 505	
Hypertriglyceridemia [*n* (%)]	No	67 (97.1)	60 (96.8)	367 (98.1)		0.602
	Sì	2 (2.9)	2 (3.2)	7 (1.9)	11 (2.2)	
		*n* = 70	*n* = 61	*n* = 370	*n* = 501	0.841
Diabetes [*n* (%)]	No	69 (98.6)	61 (100)	364 (98.4)		
	Sì	1 (1.4)	(0)	6 (1.6)	7 (1.4)	
		*n* = 56	*n* = 50	*n* = 304	*n* = 410	0.999
Abdominal obesity [*n* (%)]	No	56 (100)	50 (100)	302 (99.3)		
	Sì	(0)	(0)	2 (0.7)	2 (0.5)	
		*n* = 77	*n* = 68	*n* = 393	*n* = 538	< 0.001
Hypertension [*n* (%)]	No	69 (89.6)	68(100)	333 (84.7)		
	Sì	8 (10.4)	(0)	60 (15.3)	68 (12.6)	
		*n* = 68	*n* = 60	*n* = 358	*n* = 486	0.707
Metabolic syndrome [*n* (%)]	No	67 (98.5)	60 (100)	355 (99.2)		
	Sì	1 (1.5)	(0)	3 (0.8)	4 (0.8)	

*p* values refer to Fisher exact test

Atopic comorbidities showed a significant association to adult AD severity, with frequencies increasing from mild to moderate and severe adult AD (asthma *p* < 0.001; conjunctivitis *p* < 0.001; rhinitis *p* = 0.002; alopecia *p* < 0.001). Similarly, sleep disorders, were more frequent among patients suffering from severe, rather than mild or moderate adult AD (*p* < 0.001) (Table 3).

**Table 3:** Atopic comorbidities according to adult AD severity

		Adult AD				
		Mild	Moderate	Severe		*p*
		*n* = 79	*n* = 71	*n* = 534	*n* = 684	
Asthma [*n* (%)]	No	62 (78.5)	53 (74.6)	336 (62.9)		< 0.001
	Yes	17 (21.5)	18 (25.4)	198 (37.1)	233 (34.1)	
Conjunctivitis [*n* (%)]	No	58 (73.4)	50 (70.4)	290 (54.3)		< 0.001
	Yes	21 (26.6)	21 (29.6)	244 (45.7)	286 (41.8)	
Rhinitis [*n* (%)]	No	46 (58.2)	35 (49.3)	207 (38.8)		0.002
	Yes	33 (41.8)	36 (50.7)	327 (61.2)	396 (57.9)	
Sleep disorders [*n* (%)]	No	64 (81)	43 (60.6)	163 (30.5)		< 0.001
	Yes	15 (19)	28 (39.4)	371 (69.5)	414 (60.5)	
		*n* = 67	*n* = 51	*n* = 470	*n* = 588	
Other disturbs [*n* (%)]	No	64 (95.5)	50 (98)	428 (91.1)		0.136
	Yes	3 (4.5)	1 (2)	42 (8.9)	46 (7.8)	
		*n* = 67	*n* = 51	*n* = 469	*n* = 587	< 0.001
Alopecia [*n* (%)]	No	65 (97)	37 (72.5)	431 (91.9)		
	Yes	2 (3)	14 (27.5)	38 (8.1)	54 (9.2)	

*p* values refer to Fisher exact test

Disease duration was significantly longer in the presence of all the atopic comorbidities (Table 4), whereas among metabolic comorbidities, only hypercholesterolemia was associated with a longer disease duration.

**Table 4:** Disease duration (years) according to atopic and metabolic comorbidities

	Absent	Present	*p*
Atopic comorbidities			
Asthma	20 (10–30)	26 (17–37)	< 0.001
Conjunctivitis	20 (9–30)	25 (17–36)	< 0.001
Rhinitis	20 (8–30)	24 (15–34)	< 0.001
Metabolic comorbidities			
Hypercholesterolemia	20 (10–29)	24 (10–40)	0.039
Hypertension	20 (10–29)	27 (8.5–40)	0.182
Hypertriglyceridemia	20 (10–30)	30 (14–53)	0.111

Values are medians (1st–3rd quartiles). *p* values refer to Wilcoxon rank sum test

As regards topical treatments (Table 5), the majority part of patients (98.3%) was under treatment or had received topical corticosteroids, at least once in their life (98.7% of patients with mild adult AD, 97.1% of patients with moderate adult AD, and 98.5% of patients with severe adult AD had experience of treatment with topical corticosteroids) with an increasing trend, moving from mild to moderate and severe AD (*p* = 0.003).

**Table 5:** Topical and systemic treatments according to disease severity

		Adult AD			
		Mild	Moderate	Severe	*p*
		*n* = 79	*n* = 71	*n* = 534	
Topical corticosteroids [*n* (%)]	Never	1 (1.3)	2 (2.8)	8 (1.5)	0.003
	Previous	58 (73.4)	41 (57.7)	276 (51.7)	
	On-going	20 (25.3)	28 (39.4)	250 (46.8)	
Topical calcineurin inhibitors	Never	50 (63.3)	40 (56.3)	135 (25.3)	< 0.001
[*n* (%)]	Previous	21 (26.6)	21 (29.6)	287 (53.7)	
	On-going	8 (10.1)	10 (14.1)	112 (21)	
Systemic corticosteroids [*n* (%)]	Never	26 (32.9)	20 (28.2)	80 (15)	< 0.001
	Previous	50 (63.3)	48 (67.6)	404 (75.7)	
	On-going	3 (3.8)	3 (4.2)	50 (9.4)	
Systemic antihistamines [*n* (%)]	Never	12 (15.2)	13 (18.3)	30 (5.6)	< 0.001
	Previous	52 (65.8)	43 (60.6)	360 (67.4)	
	On-going	15 (19)	15 (21.1)	144 (27)	
		*n* = 79	*n* = 71	*n* = 531	
Puva/uva/uvbnb [*n* (%)]	Never	65 (82.3)	59 (83.1)	297 (55.9)	< 0.001
	Previous	11 (13.9)	10 (14.1)	218 (41.1)	
	On-going	3 (3.8)	2 (2.8)	16 (3)	
Cyclosporine A [*n* (%)]	Never	49 (62)	46 (64.8)	149 (27.9)	< 0.001
	Previous	27 (34.2)	18 (25.4)	329 (61.6)	
	On-going	3 (3.8)	7 (9.9)	56 (10.5)	
Metotrexate [*n* (%)]	Never	77 (97.5)	67 (94.4)	475 (89)	0.106
	Previous	2 (2.5)	4 (5.6)	52 (9.7)	
	On-going	0 (0)	0 (0)	7 (1.3)	
Azathioprine [*n* (%)]	Never	79 (100)	69 (97.2)	510 (95.5)	0.386
	Previous	0 (0)	2 (2.8)	22 (4.1)	
	On-going	0 (0)	0 (0)	2 (0.4)	
Mycophenolate mofetil/sodium [*n* (%)]	Never	79 (100)	71 (100)	533 (99.8)	0.386
	Previous	0 (0)	0 (0)	1 (0.2)	

Fisher exact test

The use of topical calcineurin inhibitors was less frequent than corticosteroids (only 32.8% of the patients was receiving or had received them in the past), among them 63.3% had mild, 56.3% moderate, and 25.3% had severe adult AD.

The trend of resorting to calcineurin inhibitors increased with the worsening of disease (*p* < 0.001). Over half of the patients (61.5%), regardless disease severity, had not received any phototherapy treatment (PUVA, UVA, UVB, UVB nb), for all the duration of their disease (82.3% of patients with mild, 83.1% with moderate and 55.9% with severe adult AD). The use of phototherapy was significantly more frequent in patients with severe adult AD, compared to those with mild or moderate adult AD (*p* < 0.001).

For what concerns to systemic treatments (Table 4), the majority of patients (81.5%) had been treated with systemic corticosteroids in the past, or they were still on treatment (67.1% of patients with mild, 71.8% moderate, and 85.1% severe AD), the use of systemic corticosteroids increases in parallel with the worsening of disease (*p* < 0.001).

Conversely, the majority of patients with mild (62%) and moderate (64.8) adult AD, had not received any cyclosporine treatment throughout their disease duration, whereas 72.1% of patients with severe adult AD had been treated in the past or they were still in treatment with cyclosporine. Thus, the use of cyclosporine was significantly more frequent in patients with severe adult AD, compared to mild and moderate adult AD (*p* < 0.001).

In general, long-term treatment with oral immunosuppressive therapy was usually introduced when topical treatment with mid- to high-potent corticosteroids and/or calcineurin inhibitors had not been successful. Among immunosuppressive drugs, Cyclosporine was the most widely used agent, for a minimum treatment period of 3 months and no longer than 2 years. Median period of cyclosporin treatment was 6.5 ± 2.8 month. Main reasons for cyclosporin discontinuation included: disease control in 25.6% of patients, adverse events in 21.2% of patients, ineffectiveness in 17.1%, and adverse events plus ineffectiveness in 5.7% of enrolled patients.

As regards other immunosuppressive agents: 97.5% of patients with mild, 94.4% of patients with moderate, and 89% of patients with severe had never been treated with methotrexate; 100% of patients with mild, 97.7% of patients with moderate, and 95.5% of patients with mild had not received azathioprine; only one patient with severe disease had had experience of treatment with mycophenolate mofetil. No difference among categories of disease severity was evident for use of immunosuppressive agents other than cyclosporine (*p* = 0.386).

Table 6 shows the results of logistic regression analysis. Disease duration and hypertension were found significantly associated with severe adult AD. In particular, the risk of having a severe adult AD increased of 2% for every year of disease duration added, and 3.52 time in presence of hypertension.

**Table 6:** Factors associated to adult AD severity

Factors	OR	95%CI	*p*
Disease duration (years)	1.02	1.01–1.04	0.006
Asthma (yes vs no)	1.41	0.82–2.47	0.215
Conjunctivitis (yes vs no)	1.56	0.87–2.81	0.135
Rhinitis (yes vs no)	1.05	0.61–1.83	0.853
Hypercholesterolemia (yes vs no)	0.66	0.40–1.10	0.108
Hypertension (yes vs no)	3.52	1.62–8.85	0.003

Results from logistic regression. LR test: *χ*^2^ = 30.7, df = 6, *p* value < 0.001; HL test:*χ*^2^ = 6.98, df = 8, *p* value 0.539

## Discussion

In the last decade, the relationships between chronic cutaneous and systemic diseases have emerged as major clinical, public health and research issues. Consequently, clinical and epidemiological researches focusing on comorbidities of skin diseases are currently recognized as one of the most important tools to indirectly increase knowledge on their physiopathology and to profile the burden of disease.

The most notable finding of our study is the confirmation of a significant association between atopic diseases like asthma, conjunctivitis, alopecia and rhinitis, and adult AD severity in Italian population through a nationwide study.

This is consistent with several data recently merging from the literature. In 2019, Kok et al. [7] reported that a dose-dependent effect can be found between the association of atopic comorbidities and severity of adult AD, which suggests that chronic severe adult AD may result in increased disease burden and morbidity.

Similarly, Sicras-Mainar A et al. [14] in 2019 described a close association between adult AD severity and other immune-allergic expressions such as asthma and rhinitis.

These data must be interpreted in the perspective that the overall prognosis for patients with one or more atopic comorbidities has a worse clinical course compared to patients suffering from atopic dermatitis alone [8, 9].

This evidence is reinforced by the observation of Thijs et al. [15] who reported that adult AD with coexistent atopic conditions is associated with more severe and extensive disease. The authors explain these associations through the increased expression of several serum pro-inflammatory mediators like PARC, TIMP-1 and sCD14, and a great selective Th-2 cytokine inflammatory pressure in this subset of patients.

These are actually expected events, if we refer to the pathogenic model of "atopic march", a temporal development model widely used in epidemiological studies to interpret the temporal changes in the prevalence of eczema, asthma, and allergic rhinitis [16, 17].

Definitive conclusions about temporality, and causal relationship between observed associations are impossible to be drawn, as most studies reported in the literature have a cross-sectional design. However, the existence of a common inflammatory pathogenic Th-2 pathway might explain the higher correlation between severity of adult AD and other atopic conditions, compared with metabolic abnormalities, as reported also by Kok et al. [7]. Indeed recently, a unifying hypothesis of a type 2 inflammation mechanism involving T Helper 2 responses has been suggested for all the comorbid atopic conditions (i.e. atopic asthma, AD, atopic conjunctivitis) [18].

The association between adult AD and metabolic abnormalities has been postulated on the basis of the “inflammatory skin march model,” first identified in psoriasis patients with systemic inflammatory condition [19].

According to this model, Th1, Th17, and Th22 pro-inflammatory cytokines mediate their effects via binding to their own cytokine receptors and then activating several downstream pathways, driving the connection between metabolic syndrome and atopic dermatitis in adults [19]. However, association between metabolic abnormalities and adult AD has not been fully established yet [20].

The cross-sectional study of Kok et al. [7] conducted on 5007 Korean adults reported that metabolic syndrome, central obesity, and hypertriglyceridemia correlated positively with adult AD in women. However, Radtke et al. [21] in their cross-sectional analysis conducted on 37,456 patients with psoriasis, and 48,140 patients with adult AD demonstrated that, unlike the psoriasis cohort, the prevalence ratios for hypertension, hyperlipidaemia, and diabetes showed no difference in adult AD patients compared to non-adult AD patients.

Moreover, a recent systematic review by Ali et al. [22], which included 14 studies elucidating the significance of metabolic comorbidities in adult AD reported a positive association between adult AD and central obesity measured as waist circumference, and this association was stronger for women than men. The association between adult AD and hyperglycaemia appears unlikely, and inconsistent for hypercholesterolemia. Non-conclusive results can be traced for hypertension. However, the associations between hypertension, hyperglycaemia, cholesterol levels, and adult AD remain unclear, and central obesity could be the only component that correlates positively with Adult AD [21]. Moreover, other data from literature confirm obesity as the only metabolic parameter to be associated with increased prevalence and severity of Adult AD [23, 24].

Finally, Thyssen JP et al. [25] analyzed the 16 most relevant studies comparing cardiovascular risk factors and diabetes for adult patients with and without AD. No association was found between adult AD and type 2 diabetes, and hypertension. The authors conclude that it is unlikely that adult AD is, in itself, a risk factor for CVD [25].

In conclusion, it remains unclear whether adult AD is a risk factor for metabolic syndrome.

Our results show that hypertension is more frequent in severe adult AD patients and suffering from hypertension increases the risk of having a severe adult AD of more than 3.5 times. Moreover, in our population, very few people were found to be obese. This is not unexpected and confirms data from a recent systematic and meta-analysis review by Ascott et al. [26] who reported that significant associations with cardiovascular outcomes and adult AD were described in cohort studies, although no evidence was found among cross-sectional studies.

Moreover, observed associations between adult AD and cardiovascular diseases might have been confounded by poor health behaviors of patients with adult AD, such as smoking, reduced physical activity and drinking alcohol [25].

In general, epidemiologic data on adult AD comorbidities are rare and inconclusive due to the heterogeneity of study populations in terms of the studied outcomes, and absence of a gold standard.

There is a need for further epidemiologic studies, focusing on the prevalence of metabolic comorbidities according to disease severity. It must be indeed pointed out that the most part of large population cohort or database studies may not always take into account the severity of adult AD in their analyses.

Therapeutic management of signs and symptoms of adult AD with systemic anti-inflammatory and immunosuppressive agents has been widely described [14, 27].

Our results seem to be consistent with previous publications, agreeing that corticosteroids and cyclosporine stands out as the most used drugs in moderate-to-severe forms of adult AD, among Italian Dermatologists.

Data from the reported cohort of patients reflect the Italian guidelines for the systemic treatment of AD: as systemic steroids have a largely unfavorable risk/benefit ratio for adult AD treatment, their long-term use in adult AD had not been generally recommended [12]. Short-term (up to 1–4 weeks) treatment had been considered a valid option only to treat an acute flare in severe cases of AD [12].

The only immunosuppressive agent with label indication for AD in Italy is cyclosporine, whose dosage can be easily personalized, in adults, based on the efficacy and tolerability in each individual patient [12]. Other immunosuppressive drugs, such as methotrexate and azathioprine, are used in the clinical practice though off-label, only when cyclosporine is contraindicated, not effective or not tolerated [12].

However, data on the efficacy of systemic treatments and the long-term safety of immunosuppressants are limited in adult AD, thus further studies are needed to standardize the treatment approach.

This is the first study aimed to evaluate the association of comorbidities with the severity of adult AD in Italian population. The strength of this study is the accurate selection of the sample size, consisting of a population cohort of 684 patients regularly diagnosed and followed at 23 national reference centres for atopic dermatitis. However, information retrospectively collected from medical records is measurably less accurate than information prospectively obtained, and for certain variables (e.g. abdominal obesity) about 30% of data were not available.

However, main limitation of our study consists in the retrospective nature of data collection, that is based on existing data recorded for reasons other than research. In this regard, it would have been of great interest to evaluate other comorbidities than those reported, unfortunately data focusing on lymphomas and other than hypertension cardiovascular diseases are not available. Similarly, incidence of use for each different UV and duration of treatment could be of interest, reflecting different approach in treatment of AD across different Italian regions. Unfortunately, details are not available in the reported cohort, and further observations in this field are warranted.

Moreover, in our case series, dupilumab was not included into the drugs evaluated, as it was not licensed and reimbursed by the Italian National Health System, at the moment of case collection. However, in the past few months, Dupilumab entered the therapeutic armamentarium of atopic dermatitis and other type 2 inflammatory disease. Many clinical trials on other biological agents and small molecules that may revolutionize the evolution and treatment of adult AD are on-going. Further studies are needed to define in more detail the clinical rationale behind the association between metabolic diseases and AD, and to evaluate the potential effect of treatments in preventing comorbidities development in adult AD.
